# Prevention of cancer-therapy related cardiac dysfunction

**DOI:** 10.1007/s11897-025-00697-x

**Published:** 2025-02-19

**Authors:** Elias Haj-Yehia, Lars Michel, Raluca I. Mincu, Tienush Rassaf, Matthias Totzeck

**Affiliations:** Department of Cardiology and Vascular Medicine, West German Heart and Vascular Center Essen, University Hospital Essen, Hufelandstraße 55, 45147 Essen, Germany

**Keywords:** Cancer, Cancer therapy-related cardiac dysfunction, Cardio-oncology, Prevention, Cardioprotection, Risk stratification

## Abstract

**Purpose of Review:**

Introduction of modern cancer therapies has led to increased survival of affected patients. With this advantage, the incidence of cancer therapy-related cardiac dysfunction (CTRCD) has increased and reasonable prevention strategies become necessary. This review outlines the major approaches to limit development and progression of CTRCD.

**Recent Findings:**

A broad range of cancer therapies can provoke CTRCD ranging from mild asymptomatic forms to severe heart failure. Profound cardiological assessment of cardiovascular comorbidities before initiation of cancer therapy allows identification of cancer patients at higher risk developing CTRCD which may also require closer surveillance. Cardioprotective adjustment of cancer therapy and initiation of cardioprotective medication and lifestyle optimization prior to anti-cancer treatment additionally limit the risk of CTRCD. During therapy, regular examination of cancer patients using high-sensitive cardiological diagnostic tools as three-dimensional (3D) echocardiography and global longitudinal strain (GLS) enables early detection of mild forms of CTRCD. This allows appropriate adjustment of cancer therapy and initiation of CTRCD treatment to prevent further progression to severe forms.

**Summary:**

Cardiological risk stratification before treatment initiation, cardioprotective interventions before and during cancer therapy, along with regular monitoring of treated cancer patients, can help prevent the development of CTRCD. This maintains the antitumor effects of cancer therapy while limiting cardiotoxic side effects resulting in improved quality of life and mortality of affected cancer patients.

## Introduction

Over the past decade, cancer survivorship significantly increased due to advancements in diagnostics and therapeutic interventions [1]. The continual evolution of early detection methods, precision oncology and the introduction of targeted anti-cancer drugs have substantially elevated the success rates of cancer treatment regimens [2, 3]. While these developments represent a breakthrough in the field oncology, they concurrently amplify the potential risk of cancer therapy-related cardiac dysfunction (CTRCD) among affected patients [4]. This condition not only compromises physical resilience but also detrimentally impacts the overall quality of life for patients undergoing cancer therapy [5]. It becomes evident that a detailed risk stratification is essential both before the initiation of cancer therapy and throughout the follow-up period [4]. Recent studies highlighted the complex interplay between cardiovascular conditions and cancer diseases, underscoring the importance of interdisciplinary cooperation of oncologists and cardiologists in cancer treatment plans [6]. Moreover, promising new biomarkers were explored holding the opportunity to identify individuals at a higher risk of developing CTRCD [4, 7, 8]. In this review, we aim to delineate the state-of-the-art strategies employed for assessment and management of risk factors associated with CTRCD to improve cardiac health in the context of cancer therapy.

### Definition of Cancer Therapy-Related Cardiac Dysfunction

CTRCD is a prominent cardiovascular complication caused by a plethora of forms of anti-cancer therapy. Radiotherapy is able to provoke CTRCD [9, 10]. This depends especially on the radiation dose to the heart and cardiac substructures (> 2 Gy/day per fraction) [11]. The risk also increases at younger age at time of radiation treatment [12, 13]. Bone marrow transplantation, notably allogenic donation, can provoke a graft-versus-host-disease (GVHD) leading to cardiac dysfunction [14, 15]. The spectrum of medicinal cancer treatments provoking CTRCD spans chemotherapy, such as anthracyclines, and protein kinase inhibitors targeting vascular endothelial growth factor (VEGF), RAF, or MEK [16–19]. Additionally, immune checkpoint inhibitors (ICI), widely employed across diverse cancer entities, contribute to the landscape of potential CTRCD triggers [20]. HER2-targeted therapies, prevalent in breast and gastric cancer, BCR-ABL inhibitors utilized for chronic myeloid leukemia (CML), and multiple myeloma therapies involving immunomodulatory drugs (IMID) and proteasome inhibitors are also able to provoke CTRCD [21–24]. Moreover, modern anti-cancer approaches hold potential risk of provoking CTRCD. Chimeric antigen receptor (CAR)-T cell therapy can trigger a systemic inflammatory response emerging as cytokine release syndrome, which leads to severe cardiac dysfunction [25].

**Fig. 1:**
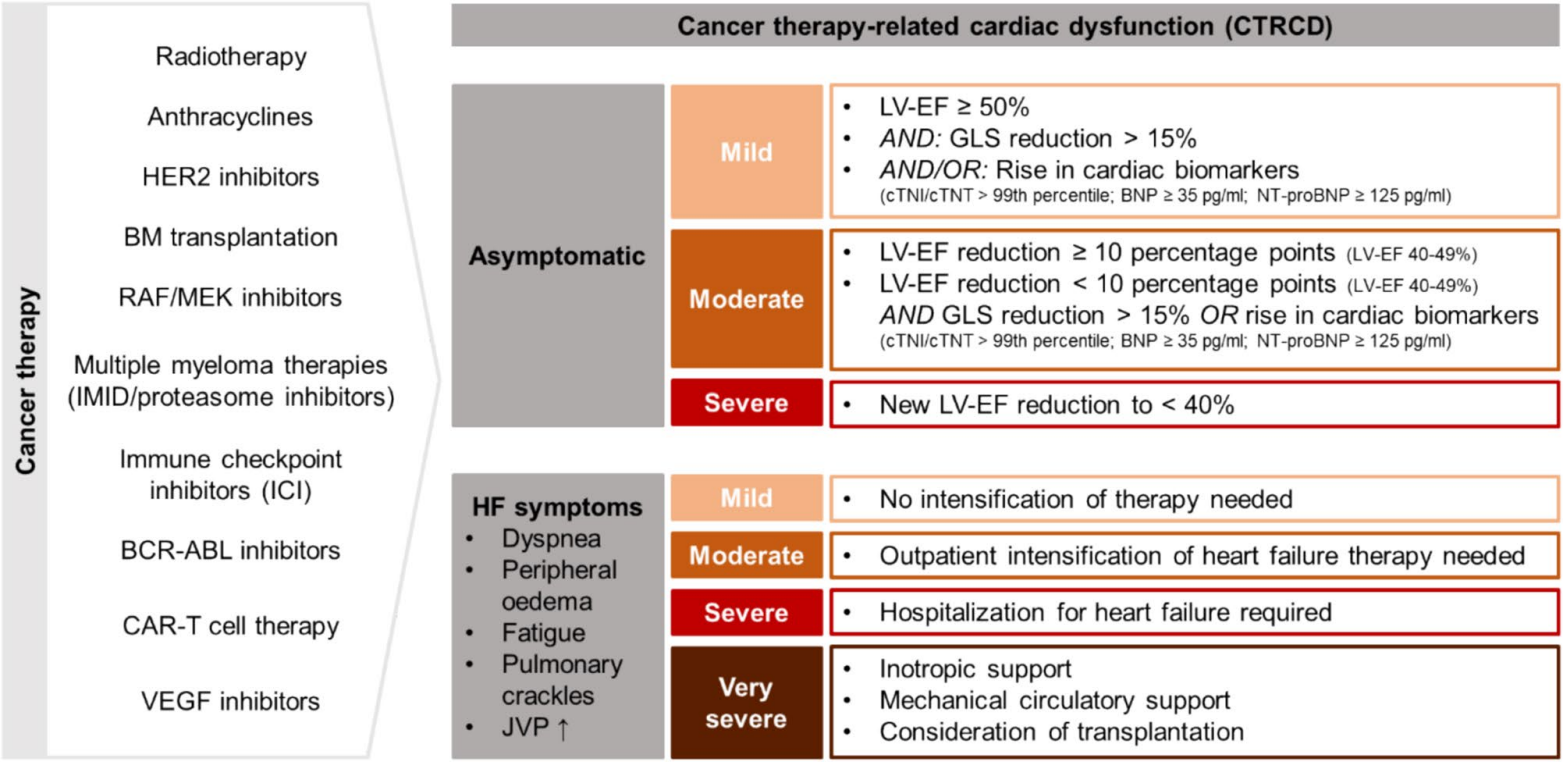
Cancer therapy provoking cancer therapy-related cardiac dysfunction (CTRCD). CTRCD occurs either asymptomatic or with heart failure (HF) symptoms. Asymptomatic CTRCD is classified into three level of severity (mild, moderate and severe) depending on reduction in left ventricular ejection fraction (LV-EF) and global longitudinal strain (GLS) or rise in cardiac biomarkers. Symptomatic CTRCD is graded by extent of therapy intensification into four level of severity (mild, moderate, severe and very severe). BCR-ABL, breakpoint cluster region-Abelson oncogene locus. BM, bone marrow. BNP, brain natriuretic peptide. CAR, chimeric antigen receptor. cTNI/cTNT, cardiac troponin I/T. HER2, human epidermal growth factor receptor 2. ICI, immune checkpoint inhibitor. IMID, immunomodulatory drug. JVP, jugular venous pressure. MEK, mitogen-activated extracellular signal-regulated kinase. NT-proBNP, N-terminal pro-B-type natriuretic peptide. RAF, rapidly accelerated fibrosarcoma. VEGF, vascular endothelial growth factor. Figure modified from Lyon et al., European Heart Journal, 2022

In 2022, the task force on cardio-oncology of the European Society of Cardiology (ESC) formulated the first ESC guidelines on cardio-oncology, thereby establishing a standardized definition of CTRCD [4]. Within the spectrum of asymptomatic CTRCD, the ESC guidelines differentiate three levels of severity (Fig. 1). Mild asymptomatic CTRCD is identified by a preserved left ventricular ejection fraction (LV-EF), coupled with a new relative decline in global longitudinal strain (GLS) exceeding 15% from baseline and/or an increase in cardiac biomarkers. In this context an increase of cardiac troponin I or T above the 99th percentile or levels of brain natriuretic peptide (BNP) ≥ 35 pg/ml and N-terminal pro-B-type natriuretic peptide (NT-proBNP) ≥ 125 pg/ml respectively are defined as a significant rise of cardiac biomarkers. Moderate asymptomatic CTRCD is characterized by a new reduction of LV-EF by more than ten percentage points to a LV-EF of 40–49%, or a new LV-EF reduction by less than ten points to an LV-EF of 40–49%, accompanied by a relative decline of GLS exceeding 15% or a rise in cardiac biomarkers. Severe asymptomatic CTRCD is present when there is a new LV-EF reduction to below 40%.

Conversely, the symptomatic manifestation of CTRCD is categorized into four severity levels (Fig. 1) [4]. No symptoms exclusive to CTRCD exist, as affected patients typically exhibit symptoms akin to those seen in heart failure resulting from other etiologies [26]. Mild symptomatic CTRCD entails heart failure symptoms not necessitating therapy intensification. In cases of moderate symptomatic CTRCD, outpatient intensification of heart failure therapy is required, while severe symptomatic CTRCD mandates hospitalization. Very severe symptomatic CTRCD reaches a critical stage, demanding inotropic and mechanical circulatory support or even prompting consideration for heart transplantation.

### Risk Stratification Before Initiation of Cancer Therapy

**Fig. 2:**
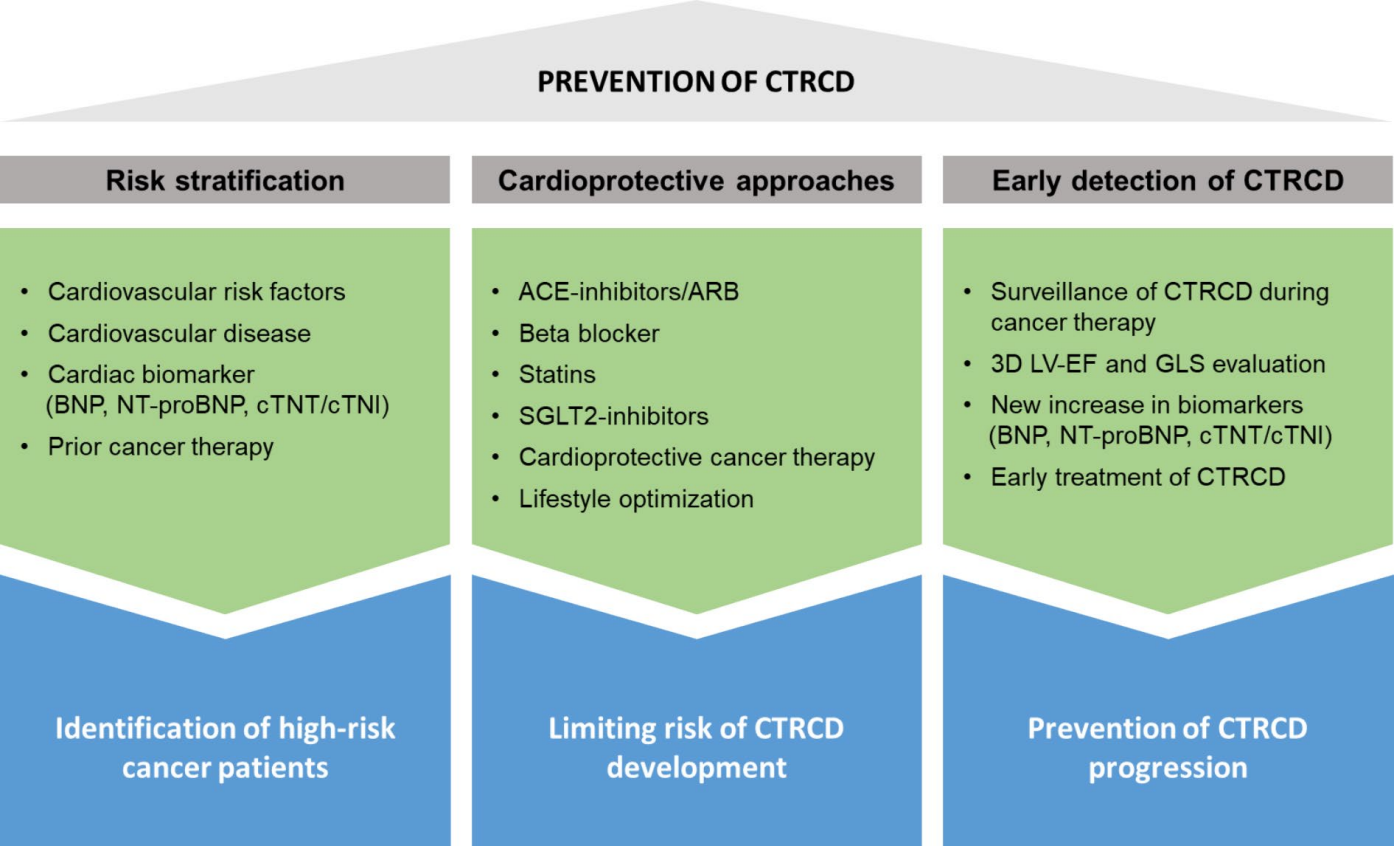
Prevention of cancer therapy-related cardiac dysfunction (CTRCD) consists of three major aspects. Risk stratification identifies cancer patients at higher risk developing CTRCD needing closer cardiologic surveillance protocols. Cardioprotective approaches, including pharmacologic and non-pharmacologic strategies, can limit the risk developing CTRCD after initiation of cancer therapy. Using high-sensitive non-invasive cardiologic diagnostic surveillance during cancer therapy enables early detection of mild forms of CTRCD. This allows adjustment of cancer therapy and initiation of CTRCD treatment preventing further progression to severe forms. ACE, angiotensin-converting enzyme. ARB, angiotensin receptor blockers. BNP, brain natriuretic peptide. cTNT/cTNI, cardiac troponin I/T. GLS, global longitudinal strain. LV-EF, left ventricular ejection fraction. NT-proBNP, N-terminal pro-B-type natriuretic peptide. SGLT-2, sodium glucose transporter 2

Prevention of CTRCD is ideally commenced at the time of cancer diagnosis. It involves a multifaceted approach, considering various aspects to assess the baseline risk of CTRCD in cancer patients (Fig. 2). These following aspects are summarized by a risk score proposed by the Heart Failure Association (HFA) and the International Cardio-Oncology Society (ICOS) to graduate the pre-treatment risk of cardiotoxic side effects under cancer therapy from low to very high [4, 27]. It is recommended to thoroughly evaluate the patient's history of cardiovascular risk factors, existing cardiovascular diseases, and prior cancer treatments [4]. This should be complemented with a series of cardiac diagnostics, including an electrocardiogram (ECG), echocardiography, and the measurement of cardiac biomarkers [4]. These diagnostic tools provide valuable insights into the baseline cardiac health of the patient, aiding in the early identification of potential risks associated with cancer therapy or undiagnosed cardiovascular disease. The HFA-ICOS risk score also places more weight on the different risk factors for the overall patient’s risk depending on the type of cancer therapy to be initiated [4, 27].

The CTRCD risk depends on age of the patients when receiving cancer treatment. People older than 60 years are endangered to develop CTRCD under therapy with anthracyclines or HER2-targeted therapy [28]. In patients older than 75 years, ICI therapy holds increased risk provoking CTRCD [4, 29]. The risk for cardiac complication in the context of bone marrow transplantation also increases with age [4]. Some cancer treatments are more detrimental when implemented at younger age. Radiotherapy or treatment with anthracyclines hold higher risk for CTRCD in younger patients [12, 13]. A meta-analysis revealed patients younger than 55 years to be more vulnerable for developing CTRCD under therapy with RAF or MEK inhibitors [19]. Besides age, sex is also a risk factor for CTRCD in some classes of cancer drugs, as female patients are at higher risk under treatment with anthracyclines [30].

Traditional cardiovascular risk factors should be reviewed as they potentiate the risk of developing CTRCD [4, 31]. This includes hypertension, diabetes mellitus, dyslipidemia or smoking. In cancer patients with unknown cardiovascular risk profile, assessment of parameters like hemoglobin A1c or blood lipids, especially low density lipoprotein cholesterol (LDL-C), might help to unmask so far undiscovered cardiovascular risk factors in these patients. Risk calculation using SCORE2 and SCORE2-OP tables represents an additional helpful tool to assess the risk level in cancer patients, although these scores are focused on patients with cardiovascular disease in general [32]. Measurement of BNP and NT-proBNP can help to assess baseline cardiovascular risk as a certain predictive capacity for future CTRCD is reported in several studies [33–35]. Cardiac troponin levels however show only limited prognostic value in patients treated with anthracyclines or HER2-targeted therapy [36]. To identify vulnerable subgroups of cancer patients at higher risk of developing CTRCD different novel biomarkers are reported for risk stratification. In patients treated with ICI therapy neutrophil-to-lymphocyte ratio (NLR) is associated with CTRCD and major adverse cardiac events (MACE) [37]. Changes in several biomarkers like myeloperoxidase (MPO), galectin-3 or C-reactive protein (CRP) among others may help predict subsequent CTRCD in breast cancer patients [38, 39]. In patients treated with doxorubicin and trastuzumab baseline levels of immunoglobin E (IgE) may represent a marker for CTRCD [40]. However, there is currently no evidence supporting routine measurement of these biomarkers and further investigations are required.

Concomitant cardiovascular diseases, e.g. heart failure, coronary artery disease, previous myocardial infarction or severe valvular heart disease, enhance the risk of developing CTRCD to a great extend [4, 41]. In these patients, additional examinations, e.g. stress echocardiography, cardiac magnetic resonance (CMR), coronary computed tomography angiography (CCTA) or nuclear perfusion imaging, are helpful to determine the overall risk status [4]. Baseline cardiologic assessment is recommended in cancer patients with or without pre-existing cardiovascular disease to either reveal potential undiagnosed cardiovascular disease and initiate corresponding therapy or optimize cardiovascular risk before start of anti-cancer treatment [4]. These approaches can help decreasing the risk of developing CTRCD during cancer therapy in these patients.

Prior exposure to specific cancer treatments, particularly anthracyclines or HER2-targeting therapies, additionally heightens the risk of developing CTRCD [4, 42]. Recognizing this increased risk prompts the need for a more vigilant oncology follow-up and necessitates additional cardiological evaluation. In such cases, the collaborative effort between oncologists and cardiologists becomes pivotal. Cancer patients at high-risk and very high-risk of developing CTRCD need an additional cardiologic referral before initiation of anti-cancer therapy [4]. This interdisciplinary approach allows for a more personalized and tailored strategy, ensuring that the therapeutic benefits are maximized while minimizing the potential cardiac complications.

In the context of an increasing personalization of cancer therapy, genetic testing gains more attention for prevention of CTRCD [43]. Advances in clinical genome sequencing revealed several candidate genes and single nucleotide polymorphisms associated with CTRCD under different anti-cancer treatments [44–47]. These genetic variants affect different aspects like transport and metabolism of anti-cancer drugs, oxidative stress response or myocardial vulnerability [4]. There are also reports indicating that not only germline genetic predispositions but also somatic mutations in the tumor itself can contribute to CTRCD [48]. So far, routine use of genetic testing to assess the risk of CTRCD before initiation of cancer therapy is currently not recommended. Nevertheless, this approach might help to define the individual vulnerability developing CTRCD during anti-cancer treatment.

### Cardioprotective Approaches During Cancer Therapy

Primary prevention of CTRCD includes different pharmacological and non-pharmacological strategies (Fig. 2). In general the use of cardiotoxic drugs and there dosage should be limited to the lowest demanding level [4, 49]. In patients under therapy with anthracyclines, HER2-targeted therapies or other chemotherapies concomitant treatment with ACE-inhibitors or ARB reduced decline in LV-EF several small randomized controlled trials [50–56]. Beta blockers and mineralocorticoid receptor antagonists are also reported to reduce CTRCD in these patients [53, 57]. Besides their lipid lowering effects statins also evolve pleiotropic anti-oxidative and anti-inflammatory effects [58, 59]. In cancer patients use of statins during anthracycline therapy led to a smaller decrease in LV-EF [60]. There is also limited data showing capability of sodium-glucose cotransporter 2 (SGLT-2) inhibitors to prevent CTRCD [61–63]. Only in patients with bladder cancer administration of SGLT-2 inhibitors might be applied with caution as some studies observed increased numbers of this cancer entity under SGLT-2 inhibitor therapy in diabetic patients [64]. However, current data on this topic remains controversial [65]. All these studies addressing SGLT-2 inhibitor treatment for prevention of CTRCD mostly included a rather small number of patients with low baseline risk for developing CTRCD. Therefore larger trials for cancer patients at higher risk are needed.

Depending on the type of cancer therapy, some specific approaches to limit the risk of CTRCD are reported. In case of radiation therapy modern technical advantages exist to improve targeting radiation delivery and minimizing the mean heart dose [66, 67]. Intensity-modulated radiation therapy or respiratory management using gating or breath-hold enable a more precise shaping of dose distribution [68, 69]. Other modalities like proton therapy are able to further decrease exposure to surrounding healthy organs during radiation therapy [10].

In cancer patients treated with anthracyclines or HER2-targeting therapies dexrazoxane reduces development of CTRCD [70, 71]. This derivative of ethylenediaminetetraacetic acid (EDTA) acts as an iron chelator diminishing formation of active anthracycline metabolite complexes and interaction between topoisomerase II and anthracycline [72]. This prevents cardiotoxicity by reducing oxidative stress and DNA damage [73]. This therapy is so far approved in adult patients with advanced or metastatic breast cancer receiving a minimum cumulative anthracycline dose of 300 mg/m^2^ doxorubicin or equivalent [4]. It should be considered in patients treated with a high total cumulative anthracycline dose and in patients at high or very high risk of developing CTRCD [4]. Of note, no reduction in antitumor efficacy was found in breast cancer patients treated with dexrazoxane [71]. Similar results are reported for liposomal anthracyclines [74]. These modified cancer drugs enable less toxic pharmacokinetics and tissue distribution while maintaining antitumor efficacy [75, 76]. So far liposomal daunorubicin and peglycated or non-peglycated liposomal doxorubicin are available [74]. Peglycated liposomal doxorubicin is approved for metastatic breast cancer, advanced ovarian cancer, multiple myeloma and immune-deficiency syndrome-related Kaposi sarcoma while non-peglycated doxorubicin is only used in breast cancer patients so far [4]. Liposomal daunorubicin is applied in patients with acute leukemia instead of daunorubicin [4]. These liposomal anthracyclines are reported to provoke less CTRCD [74–76]. Accordingly, they are used in patients with high or very high CTRCD risk during cancer therapy [4].

Besides pharmacological approaches to prevent development of CTRCD during cancer therapy, non-pharmacological strategies are reported as well. Regular exercise can lead to an improved cardiorespiratory fitness, but its ability to prevent CTRCD is unclear [77–81]. There are some studies reporting lower rates of CTRCD or cardiovascular events in breast cancer patients undergoing routine physical activity [82]. Nevertheless, it supports a healthy lifestyle in any way and should be combined with optimization of cardiovascular risk factors [83–85]. This includes smoking cessation, restriction of alcohol consumption and intensive treatment of arterial hypertension, diabetes mellitus and dyslipidemia [4].

### Early Detection of Cancer Therapy-Related Cardiac Dysfunction

During cancer therapy regular cardiologic assessments are recommended to prevent deterioration of already evolved CTRCD and its transition to severe forms [4]. The structure of the surveillance protocol differs between different cancer therapies and depends on the baseline risk for developing CTRCD in the individual patient with shorter control intervals [4]. Early detection of asymptomatic CTRCD and initiation of appropriate therapeutic counteractions might prevent transition to symptomatic CTRCD with a potentially severe progress. To achieve this, cardiovascular imaging with echocardiography as recommended first-line modality for assessment of cardiac function and CTRCD in cancer patients can be supportive [4, 86–89]. Cancer patients with reduced baseline LV-EF values < 50% are at higher risk developing future CTRCD and should be controlled more frequently during cancer therapy [4, 86]. The same applies also for patients with borderline LV-EF values (50–54%) at baseline [4, 86]. In these patients three-dimensional (3D) LV-EF and GLS assessment can provide early detection of asymptomatic myocardial injury [90, 91]. GLS evaluation shows maximized specificity with a threshold of a relative GLS decrease of more than 15% compared with baseline [92]. In patients with limited echocardiography image quality, cardiovascular magnetic resonance (CMR) imaging can be considered instead [4]. Measurement of standard cardiac biomarkers like troponin, BNP or NT-proBNP is recommended to support diagnosis of asymptomatic forms of CTRCD [4, 93]. Baseline measurement of these biomarkers can be used to identify subclinical cardiac injury by degree of change in these biomarkers during cancer therapy [94]. In case of cardiac troponin measurment incidence of CTRCD can markedly differ depending on application of sex-specific cut-off values and type of assay [95]. It must be emphasized that cancer-specific cut-off values for these cardiac biomarkers are not established yet and levels must be interpreted in the context of possible confounders like sex, age, renal function, obesity and differential diagnostic comorbidities [4].

Early detection of CTRCD can influence further therapeutic decisions leading to initiation of cardiovascular treatment or adjustment of cancer therapy [4]. Discontinuation of cancer therapy depends on the level of CTRCD severity and relies on interdisciplinary decision between oncologists and cardiologists [4, 96]. Therapy of CTRCD follows the guidelines for treatment of heart failure [26]. While angiotensin-converting enzyme (ACE) inhibitors, angiotensin receptor blockers (ARB) or beta blockers can be used for treatment of mild asymptomatic CTRCD, moderate or severe asymptomatic CTRCD and symptomatic CTRCD requires intensified heart failure therapy including mineralocorticoid receptor antagonists (MRA), neprilysin inhibition and SGLT-2 inhibitors [4, 26, 97]. Early initiation of CTRCD targeting therapy prevents progression to severe forms and allows continuation of cancer therapy with minimizing cardiovascular side effects.

## Conclusion

This review has highlighted the most important strategies to prevent development of CTRCD in cancer patients. It emphasizes the diagnostic criteria for asymptomatic and symptomatic forms of CTRCD. This helps to identify early and mild forms of CTRCD allowing appropriate cancer therapy adjustment and initiation of CTRCD treatment to prevent further progression at an early stage. Several strategies for risk stratification were described enabling identification of cancer patients at higher risk to develop CTRCD. These patients need closer cardiological surveillance protocols for early detection of possible cardiotoxic side effects under cancer therapy. Different pharmacological and non-pharmacological cardioprotective approaches decrease the risk of CTRCD development after initiation of anti-cancer treatment. These prevention strategies can reduce cardiotoxic side effects in cancer patients and thus maintain positive effects of cancer therapy by limiting the need of treatment adjustment.

## Data Availability

No datasets were generated or analysed during the current study.
